# Whole leg compression garments influence lower limb kinematics and associated muscle synergies during running

**DOI:** 10.3389/fbioe.2024.1310464

**Published:** 2024-02-20

**Authors:** Chenhao Yang, Yang Yang, Yongxin Xu, Zhenyuan Zhang, Mark Lake, Weijie Fu

**Affiliations:** ^1^ School of Exercise and Health, Shanghai University of Sport, Shanghai, China; ^2^ Key Laboratory of Exercise and Health Sciences of Ministry of Education, Shanghai University of Sport, Shanghai, China; ^3^ Research Institute for Sport and Exercise Science (RISES), Liverpool John Moores University, Liverpool, United Kingdom

**Keywords:** compression garment, muscle synergy, biomechanics, kinematics, running

## Abstract

The utilization of compression garments (CGs) has demonstrated the potential to improve athletic performance; however, the specific mechanisms underlying this enhancement remain a subject of further investigation. This study aimed to examine the impact of CGs on running mechanics and muscle synergies from a neuromuscular control perspective. Twelve adult males ran on a treadmill at 12 km/h, while data pertaining to lower limb kinematics, kinetics, and electromyography were collected under two clothing conditions: whole leg compression garments and control. The Non-negative matrix factorization algorithm was employed to extract muscle synergy during running, subsequently followed by cluster analysis and correlation analysis. The findings revealed that the CGs increased knee extension and reduced hip flexion at foot strike compared with the control condition. Moreover, CGs were found to enhance stance-phase peak knee extension, while diminishing hip flexion and maximal hip extension during the stance-phase, and the ankle kinematics remained unaltered. We extracted and classified six synergies (SYN1-6) during running and found that only five SYNs were observed after wearing CGs. CGs altered the structure of the synergies and changed muscle activation weights and durations. The current study is the first to apply muscle synergy to discuss the effect of CGs on running biomechanics. Our findings provide neuromuscular evidence for the idea of previous studies that CGs alter the coordination of muscle groups, thereby affecting kinematic characteristics during running.

## 1 Introduction

Compression garments (CGs) are extensively used in various sports. These garments apply pressure to and cover the body surface, and are speculated to offer multiple benefits to athletes. These include the modulation of heat loss, enhancement of sweat evaporation, and increased comfort during exercise (Yang, et al., 2020b). Previous studies have demonstrated that the pressure from CGs can enhance athletic performance. Several possible mechanisms have been suggested, such as reducing soft tissue vibration and muscle activation for joint stability (Valle et al., 2013; Play et al., 2022), altering proprioceptive feedback and neuromuscular control (Perlau et al., 1995; Bernhardt and Anderson, 2005) and coordination (Birmingham et al., 1998), and facilitating blood and lymph circulation and metabolite clearance (O’Riordan et al., 2023); Ibegbuna et al., 2003). These mechanisms can be interrelated and hence require further investigation.

Previous studies have examined the effects of CGs on the kinematic parameters, but these studies have produced inconsistent results. For instance, a study have reported that wearing CGs led to a reduction in hip flexion angle and an increase in stride length during sprinting (Born et al., 2014); in a study of female athletes, CGs were found to reduce only hip abduction angle in the drop vertical jump task (Zamporri and Aguinaldo, 2018). However, previous studies have not found that CGs immediately affect kinetics during activity, such as joint force or power (Wannop et al., 2016; Deng et al., 2021). Some studies found no significant performance improvement in jumping (Berry and McMurray, 1987; Ali et al., 2010), running (Vercruyssen et al., 2014), or sprinting (Atkins et al., 2020) with CGs. These inconsistent results may be due to the different types of CGs used, varying in structure, fabric, pressure, etc. While CGs did not affect muscle strength acutely, it did reduce the EMG activities and maintained similar power output during repetitive muscle contractions (Born et al., 2014; Deng et al., 2021). Researchers have therefore attempted to understand the effects of CGs in terms of proprioception and neuromuscular control. Studies have also reported neurobiomechanical evidence for improved precision of movement (Hooper et al., 2015), increased responsiveness and movement-related cortical potentials (Lee et al., 2017) when wearing CGs. The current consensus appears to indicate that the compression may not directly enhance strength or power output, but it could influence neural control through its physical benefits of pressurization, providing stability, and proprioceptive input (Engel et al., 2016; Lee et al., 2017).

During daily activity tasks, external information stimuli from the changing environment cause the central nervous system (CNS) to adjust motor commands. The CNS selects muscles and encodes information on muscle synergistic activation, which is ultimately reflected in locomotion (Alessandro et al., 2018). Bernstein (1967) defined muscle synergy as the coordination of large muscle groups. Muscle synergy theory has been used to describe how neuromuscular control is achieved in movement (Cheung et al., 2005; Bizzi and Cheung, 2013), and explain the learning, transfer and change of motor skills (Sawers et al., 2015; Matsunaga and Kaneoka, 2018). Running is one of the world’s most popular sports that is widely studied and discussed. Researchers argued that muscle synergies for running are inborn or determined early in life (Cheung et al., 2020), and thus muscle synergies in running are relatively stable at the individual level. However, the development of motor skills and the change of external conditions may still fine-tune or reshape these early synergies (Santuz et al., 2018; Mileti et al., 2020). For example, as running experience increases, specific muscle synergies coalesce to become merged synergies (Cheung et al., 2020). Different foot strike patterns for running also show different muscle synergies (Nishida et al., 2017), and the body also has different muscle synergy strategies to respond to changes in fatigue conditions before and after running (Matsunaga et al., 2017). However, muscle synergies were similar across different running surfaces (concrete vs. grass) (Yaserifar and Oliveira, 2023). It has also been found that types of synergy are consistent between level and uphill running (Saito et al., 2018). These studies suggest that muscle synergy in running is relatively stable but can be influenced under certain circumstances. These evidences give us a new perspective to explore the effects of CGs. We believe it is of interest to explore the effects of CGs on the neuromuscular control of this fundamental form of locomotion, the current study will therefore investigate how running mechanics and muscle synergies are influenced by CGs.

The current cross-sectional study aimed to investigate the acute effects of whole leg compression garments (CGs) *versus* no compression on running mechanics and muscle synergies, as evidence for a modification in neuromuscular control. Based on the current evidence, we hypothesized that whole leg CGs would alter the lower body kinematics, especially at the knee and hip joints that are covered by them, but not at the ankle joint. We also expected that the structure of the muscle synergies (e.g., the weights of the muscles in the synergy, the activation timing of the synergy) would change accordingly, which would allow us to explore their relationship. Furthermore, we did not anticipate that CGs would significantly affect the kinetic parameters of running.

## 2 Methods

### 2.1 Participants

A total of 12 healthy physically active adult males (age: 23.3 ± 2.1 years; height: 177.2 ± 6.6 cm; weight: 73.3 ± 5.7 kg; weekly running bouts ≥2) were recruited for this study (Vercruyssen et al., 2017; Hsu et al., 2020). The inclusion criteria were: 1) regular physical activity of at least two sessions per week; 2) no history of musculoskeletal injury that could affect the experimental outcomes in the previous 3 months; and 3) no medical or psychological contraindications to exercise such as neuromuscular disorders. The study protocol and participant safety procedures were approved by the Ethics Committee of Shanghai University of Sport (NO. 102772021RT085). All participants provided written informed consent after being informed of the experimental aims, methods, risks, and benefits. They were also instructed to comply with the experimental requirements and procedures.

### 2.2 Experimental procedure

After confirming their voluntary participation and compliance with the inclusion criteria, the participants were provided with sport shoes and fitted with electromyography (EMG) electrodes. A random number generator was then used to ensure that the order in which they wore the two garments was randomized (1: compression garment; 2: control). Next, reflective markers were attached to their lower limbs using the same set as the previous study (Yang et al., 2019). Before the test, the participants performed stretching exercises and a 3-min warm-up at the experimental speed (12 km/h). During the test, the participants ran on a treadmill at a constant speed of 12 km/h. According to previous studies, 12 km/h is an appropriate speed setting to compare and discuss with other studies (Yang C. et al., 2020; Zhang et al., 2020). After 30 s of steady-state running, all equipments simultaneously recorded data for 15 s to ensure that at least 10 gait cycles are captured. Then, the participants changed their clothing and repeated the test under the other condition. A rest period of 5–10 min was given between the tests, and the procedure was identical to the first condition.

For the compression garment (CG) condition, we used a commercially available brand (2XU^®^ elite MA4610b) of gradient whole leg CG in size M (175/75). The manufacturer-provided compression level was approximately 15 mmHg, with a gradient distribution that decreased from the distal to the proximal end to ensure that the garment provides compression on the whole leg. However, due to individual variations in thigh circumference and curvature, the actual pressure exerted by the CG differed among participants. We have quantified the rang of compression across individuals while wearing the same type of CG, we measured the pressure at the proximal (gluteus maximus muscle) and distal (gastrocnemius muscle) ends using a single-point flexible low-pressure sensor (Novel-Q210510). The final compression level range for participants included in this study was 21.3 ± 1.4 mmHg to 12.5 ± 1.1 mmHg. The control condition involved wearing regular sport shorts without any compression. The experiment used traditional running shoes with a foam and air cushion midsole, heel-to-toe drop of 12 mm, and an average weight of 285 g, with shoe sizes ranging from 42–44.

### 2.3 Data collection

We used an eight-camera motion capture system (Vicon T40, Oxford Metrics, UK) to record the trajectory of reflective markers at a sampling rate of 200 Hz. We also used a Bertec treadmill (FIT5) instrumented with two 3D force plates (175 cm × 50 cm, Bertec^®^, USA) to measure the ground reaction force (GRF) at 1,000 Hz during running.

We used the wireless EMG system (Noraxon Ultium EMG) to record surface EMG signals during the experiment at a sampling rate of 2000 Hz. The EMG system was triggered synchronously with the motion capture system and the force plate. Prior to electrode placement, the participants’ skin was shaved and cleaned with alcohol swabs to reduce skin impedance to below 5 mega ohms. In previous studies, the number of muscles required to extract muscle synergies of unilateral lower limb ranged from 8–32 (Cappellini et al., 2006; Clark et al., 2010; Mileti et al., 2020). Therefore, we chose 9 lower limb muscles as they are primary muscles contributing to running movement and can be accurately measured by surface EMG. Electrodes were placed on the 9 muscle sites of the dominant leg (kicking leg) following the standardized placement guidelines (Noraxon^®^ 2022): gluteus maximus (GM), biceps femoris (BF), rectus femoris (RF), vastus medialis (VM), vastus lateralis (VL), tibialis anterior (TA), medial gastrocnemius (MG), lateral gastrocnemius (LG), and soleus (SO). The reflective markers and electrodes placement were performed by the same experienced experimenter to minimize error.

### 2.4 Data processing

The acquired data was post processed using Visual 3D software (v5, C-Motion, Inc., Germantown, MD, USA). A multi-segment rigid 3D model of the lower body was generated and the Helen Hayes model was applied to perform kinematic and inverse dynamic calculations. The process was automated using the software’s pipeline program. Markers trajectory and GRF data were filtered using a fourth order dual-pass Butterworth filter with cutoff frequencies of 7 Hz and 50 Hz (Yang C. et al., 2020). Joint angles, angular velocities, moments, and power of the hip, knee, and ankle joints were calculated, the description of the parameters and definition of the joint angles refer to our previous study (Yang C. et al., 2020). The foot strike and takeoff times were identified by a threshold of vGRF greater than 10 N to define stance phase and swing phase of the gait cycle, and the peak vGRF indicating the propulsion phase initiation (Yang C. et al., 2020). To investigate the effect of CGs on vertical work during running, we further analysed the vertical stiffness (k = GRF_i_/∆y) and vertical energy loss during stance phase (Liu et al., 2006; Cheung et al., 2020). GRF_i_ represents the vertical GRF when the center of gravity (CoG) was lowest, and ∆y represents the vertical displacement of CoG during centrifugation; the vertical energy loss is defined as the difference between the positive and negative work applied by the vGRF to the CoG (weight normalised). EMG data were segmented into weight acceptance, propulsion, early swing and late swing phases based on the events of the kinematics and GRF data (Mileti et al., 2020). To ensure statistical robustness, ten gait cycles per participant were averaged to obtain the final parameters.

The EMG data were preprocessed as the following steps: 1) mean removal and Butterworth bandpass filtering with a cutoff frequency range of 20–400 Hz; 2) rectification and low-pass filtering at 20 Hz to obtain the EMG envelope; 3) normalization by the maximum amplitude values of each channel; and 4) resampling and averaging of 10 gait cycles to obtain an average EMG envelope for each channel. Non-negative matrix factorization (NNMF) (Cheung et al., 2020; Rabbi et al., 2020) was then applied to the EMG data to extract the muscle synergies during running. The detailed steps are as follows:

The raw EMG matrix of each participant was decomposed using Matlab r2017 according to the following equation (Cheung et al., 2020):

$$M_{t\times 9}\approx C_{t\times n}W_{n\times 9}$$ (1)

where *M* represents the raw EMG matrix, *t* represents the number of rows of normalized data, *C* represents the activation time-series curves corresponding to the muscle synergies; *W* represents the muscle synergy matrix, which has 9 columns, representing 9 muscles involved in the calculation of the activation weight for each synergy in this study (Cheung et al., 2020); *n* represents the number of linear combinations of muscle synergies.

The optimal number of synergies, denoted by *n*, was determined by comparing the variance accounted for (VAF) by the reconstructed matrix *M′* with the original matrix *M*. The VAF is calculated using the following equation:

$$VAF=1-\frac{{\left(M-M\prime \right)}^{2}}{M^{2}}$$ (2)

Where *M* is the original matrix, and *M′* is the constructed matrix from the Eq 1.

The value of *n* was determined by the criterion of VAF exceeding 90%. Since there were 9 channels of EMG signals in this study, *n* was sequentially assigned integers from 1 to 9. For each value of n, the original matrix *M* was subjected to 25 iterations of NNMF. The final synergy matrix W and activation time course *C* for the current value of n were obtained when the residual between the reconstructed matrix M′ and the original matrix *M* was minimized (Cheung et al., 2020).

The synergies were classified using k-means clustering analysis based on previous research (Mileti et al., 2020). Six synergy clusters formed by control conditions were taken as reference synergies and labeled as Synergy 1–6 (SYN1-6). The synergies of each participant under different clothing conditions were compared with the reference synergies using Pearson correlation analysis. The correlation coefficient *r* between the participant’s synergy *W* and the centroid of the clusters was computed. The synergy was classified into one category if *r* > 0.6, indicating similarity. If *W* was similar to two or more reference synergies, it was assigned to the category with the highest *r* value (Cheung et al., 2020; Mileti et al., 2020). Furthermore, according to the methodology of previous study (Zelik et al., 2014; Xiong et al., 2018), muscle synergies were defined as active if their normalized value exceeded 0.3 of maximum activation.

### 2.5 Statistics

Statistical analysis was performed using SPSS 22.0 software and all parameter values were expressed as mean ± standard deviation. The Shapiro-Wilk test was used to test the normality of all parameters. Paired sample t-tests were used to analyze the differences in biomechanical parameters between the CG and control condition (CC). However, after extracting the muscle synergies, each participant had a different number of muscle synergies, resulting in an inconsistent number of people for each synergy after categorisation (Table 2). Therefore, referring to similar situations in previous studies (Bakker and Wicherts, 2014; Hung et al., 2022), Welch t-tests performed on the muscle synergy analysis parameters (i.e., activation weight, activation duration, start and end times) between CC and CG. The significance level α) was set at 0.05.

## 3 Results

### 3.1 Kinematics and kinetics

The step frequency (CC: 171.33 ± 7.63 steps/min; CG: 172.33 ± 7.20 steps/min) and ground contact time (CC: 288.92 ± 11.34 ms; CG: 289.70 ± 18.01 ms) did not differ significantly between the two conditions.

**FIGURE 1:**
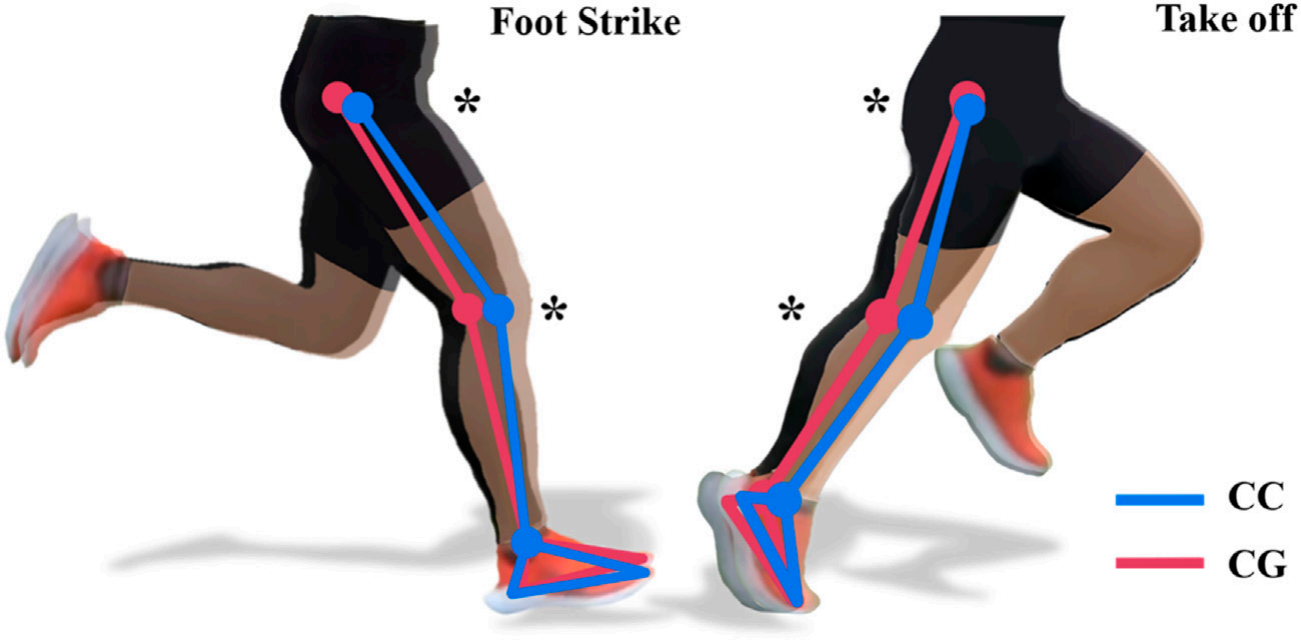
Changes in Kinematics with CG (Schematic diagram). Note: CC: Control Condition; CG: Compression Garments; the diagram shows the schematic when foot strike and take off; the image of the CC is semi-transparently placed in the upper layer and the image of the CG in the lower layer. *: Significant difference between CG and CC, *p* < 0.05.

The CGs resulted in a smaller hip flexion angle at foot strike (*p* = 0.036), a lower peak hip flexion angle (*p =* 0.014), and a higher peak hip extension angle (*p =* 0.010) during the stance phase. The knee joint angle at foot strike (*p =* 0.018) and the minimum knee joint flexion angle during the stance phase (*p =* 0.003) were also reduced by CGs. We have shown these changes schematically (Figure 1). However, it did not affect the ankle kinematics or the angular velocity of the three joints of the lower body during the stance phase (Table 1). Moreover, the compression garments condition had no effect on the kinetics of the three joints, including moment and power (Supplementary Table S1).

**TABLE 1:** Effects of compression garment on kinematics of ankle, knee and hip (mean ± SD).

*Joint*		CC	CG	*p*	*Cohen’s d*
*Ankle*	*θ*_*dor*_ (deg)	22.20 ± 2.95	23.74 ± 2.56	0.187	0.57
	*θ*_*pla*_ (deg)	−4.86 ± 3.90	−3.62 ± 3.96	0.335	0.41
	*θ*_*strike*_ 11deg)	8.75 ± 8.64	11.38 ± 5.73	0.225	0.52
	*ω*_*dor*_ (deg·s^-1^)	237.57 ± 59.61	244.28 ± 50.40	0.544	0.26
	*ω*_*pla*_ (deg·s^-1^)	−202.05 ± 46.28	−199.31 ± 53.76	0.827	0.09
	*ω*_*strike*_ (deg·s^-1^)	−169.21 ± 49.54	−145.74 ± 52.44	0.079	0.79
	*RoM* (deg)	27.05 ± 2.40	27.35 ± 3.67	0.819	0.10
*Knee*	*θ*_*min*_ (deg)	**−13.50 ± 4.47**	**−10.70 ± 4.94^a^**	**0.003**	**1.52**
	*θ*_*max*_ (deg)	−39.06 ± 4.22	−37.64 ± 3.68	0.242	0.50
	*θ*_*strike*_ (deg)	**−14.79 ± 5.41**	**−12.52 ± 6.66^a^**	**0.018**	**1.14**
	*ω*_*fle*_ (deg·s^-1^)	−97.57 ± 36.59	−91.75 ± 35.75	0.600	0.22
	*ω*_*ext*_ (deg·s^-1^)	60.00 ± 28.95	72.87 ± 62.50	0.532	0.26
	*ω*_*strike*_ (deg·s^-1^)	20.54 ± 38.73	−3.96 ± 45.09	0.102	0.73
	*RoM* (deg)	25.56 ± 3.94	26.94 ± 5.34	0.286	0.46
*Hip*	*θ*_*max*_ (deg)	**41.21 ± 9.24**	**35.32 ± 4.66^a^**	**0.014**	**1.19**
	*θ*_*min*_ (deg)	**0.96 ± 6.44**	**−4.42 ± 5.04^a^**	**0.010**	**1.27**
	*θ*_*strike*_ (deg)	**40.07 ± 9.62**	**34.68 ± 4.63^a^**	**0.036**	**0.98**
	*ω*_*fle*_ (deg·s^-1^)	62.16 ± 40.02	64.63 ± 34.42	0.856	0.08
	*ω*_*ext*_ (deg·s^-1^)	−106.66 ± 28.84	−123.39 ± 37.68	0.185	0.58
	*ω*_*strike*_ (deg·s^-1^)	25.10 ± 46.37	41.79 ± 32.48	0.341	0.41
	*RoM* (deg)	40.25 ± 4.44	39.74 ± 4.17	0.528	0.27

Note: CC: control condition; CG: compression garments; *θ*_*dor*_: peak dorsiflexion angle; *θ*_*pla*_: peak plantarflexion angle; *θ*_*min*_: minimum joint angle; *θ*_*max*_: maximum joint angle; *θ*_*strike*_: joint angle at foot strike; *RoM*: range of motion; *ω*_*dor*_: peak dorsiflexion velocity; *ω*_*pla*_: peak plantarflexion velocity. The bold values indicate the data with significant differences and are intended to highlight them.

^a^ Significant difference between CG, and CC, *p* < 0.05.

### 3.2 Muscle synergy number

**FIGURE 2:**
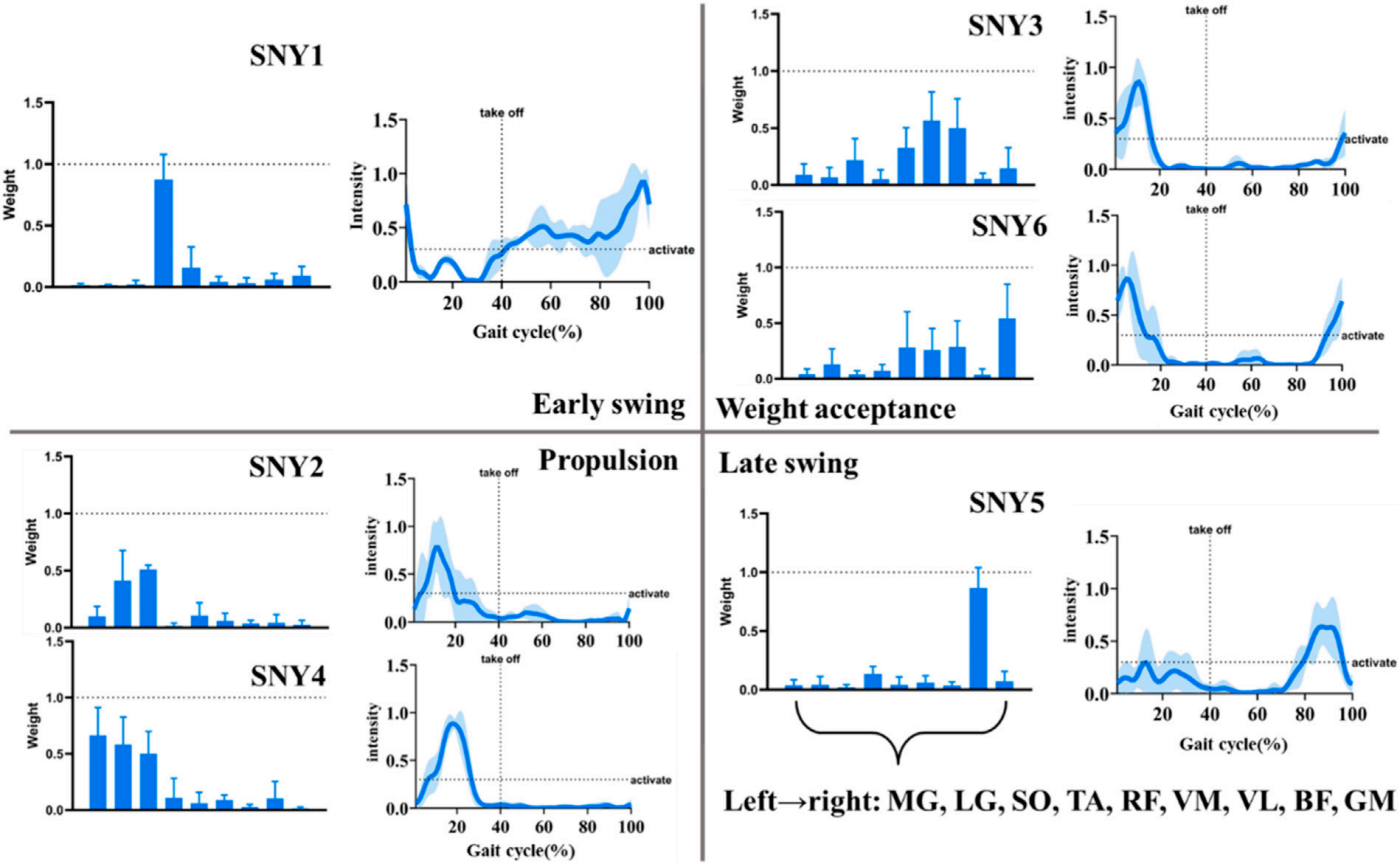
Functional schematic diagram of 6 synergies. Note: MG: Medial Gastrocnemius; LG: Lateral Gastrocnemius; SO: Soleus; TA: Tibialis Anterior; RF: Rectus Femoris; VM: Vastus Medialis; VL: Vastus Lateralis; BF: Biceps Femoris; GM: Gluteus Maximus; Shaded area is standard deviation of mean activation curve across participants.

Six muscle synergies were identified by cluster analysis of muscle synergies under control conditions. Figure 2 shows the function of these synergies during the gait cycle in the CC condition. SYN3 and SYN6 mainly provided body weight acceptance. The main muscles activated in SYN3 are the rectus femoris (RF), vastus medialis (VM), vastus lateralis (VL); in SYN6, in addition to these three, the gluteus maximus (GM) is also activated. Activation started before foot strike and peaked in the early stance phase. SYN2 and SYN4 mainly provided propulsion, the main muscles activated in SYN2 are the lateral gastrocnemius (LG) and soleus (SO), and SYN4 also includes the medial gastrocnemius (MG). Activation started at foot strike and peaked in late stance phase. SYN1 mainly provided early swing function with the main activated muscle being tibialis anterior (TA). The activation started before take-off and continued until foot strike. SYN5 mainly provided the late swing function, with the main activated muscle being biceps femoris (BF). Activation started in late swing and ended at foot strike.

As shown in Table 2, there was no difference in the average number of muscle synergies per participant between the two conditions. However, there was a difference in the distribution of synergy types. SYN2 was present in half of the participants under control conditions, but absent in all participants under CGs conditions. Conversely, SYN4 was present in all participants under CGs conditions. Moreover, some participants exhibited unmatched synergies that did not belong to any of the reference synergies.

**TABLE 2:** Number of synergies (N_SYN_).

Group	n_SYN1_	n_SYN2_	n_SYN3_	n_SYN4_	n_SYN5_	n_SYN6_	N_SYN_	n_U-M_
CC	12	6	12	9	12	9	5.75 ± 0.62	9
CG	12	0	12	12	12	8	5.25 ± 0.45	7

Note: CC: control condition; CG: compression garments; *N*_*SYN*_: average number of synergies per person; *n*_*U-M*_: number of unmatched synergies.

### 3.3 Muscle activation weights in muscle synergy

**FIGURE 3:**
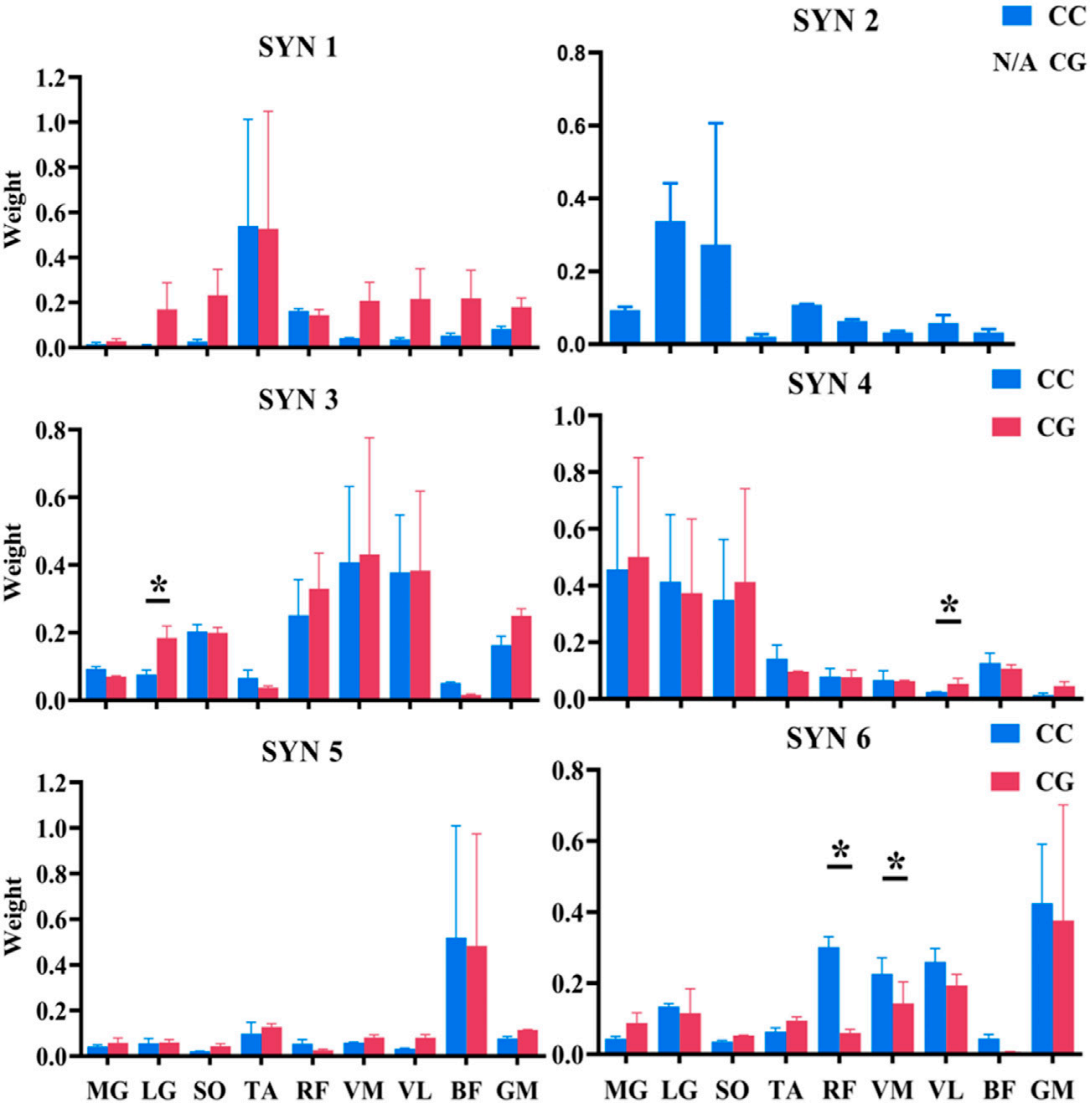
Weight of Muscles Activation in SYN (1–6). Note: CC: Control Condition; CG: Compression Garments; MG: Medial Gastrocnemius; LG: Lateral Gastrocnemius; SO: Soleus; TA: Tibialis Anterior; RF: Rectus Femoris; VM: Vastus Medialis; VL: Vastus Lateralis; BF: Biceps Femoris; GM: Gluteus Maximus; *: Significant difference between CG and CC, *p* < 0.05.

Figure 3 shows the activation weights of these synergies. There were no significant differences in the weights of each muscle activation for SYN1 and SYN5 regardless of conditions. SYN2 was absent under CG conditions. When wearing CG, the activation weight of LG (SYN3) significantly increased by 215%, while the weight of VL (SYN4) significantly increased, but the latter remained relatively low. The CGs condition decreased the activation weight of RF and VM by 76% and 61%, respectively. (Table 3; Supplementary Table S2).

**TABLE 3:** Weight of muscle activation in SYN (mean ± SD).

Muscles (%)	SYN2		SYN3		SYN4		SYN6	
	CC	CG	CC	CG	CC	CG	CC	CG
MG	9.97 ± 8.73	N/A	8.83 ± 9.75	6.86 ± 7.17	66.25 ± 24.96	74.85 ± 25.26	3.95 ± 4.83	6.90 ± 10.80
LG	41.11 ± 26.41	N/A	**6.64 ± 8.59**	**20.95 ± 16.03^a^**	58.11 ± 24.62	55.82 ± 18.90	12.87 ± 13.99	16.40 ± 6.71
SO	50.89 ± 3.74	N/A	21.83 ± 18.93	21.12 ± 18.81	49.98 ± 19.87	64.49 ± 18.14	3.79 ± 3.37	5.34 ± 5.28
TA	1.49 ± 2.56	N/A	5.01 ± 8.31	3.42 ± 4.10	10.82 ± 17.60	9.75 ± 9.48	7.12 ± 5.67	10.21 ± 8.81
RF	10.71 ± 10.95	N/A	32.67 ± 17.65	40.40 ± 25.60	5.90 ± 9.88	5.81 ± 9.47	**28.17 ± 32.19**	**6.74 ± 5.37^a^**
VM	5.99 ± 6.70	N/A	56.67 ± 24.84	67.54 ± 18.65	8.90 ± 4.37	6.14 ± 6.50	**25.82 ± 19.40**	**10.07 ± 18.58^a^**
VL	3.52 ± 2.94	N/A	49.82 ± 25.74	54.93 ± 21.66	**2.55 ± 2.42**	**6.66 ± 3.93^a^**	28.67 ± 23.41	17.09 ± 21.59
BF	4.27 ± 7.32	N/A	5.35 ± 4.96	1.44 ± 1.74	10.35 ± 15.14	9.80 ± 11.66	3.61 ± 5.20	0.68 ± 0.74
GM	2.57 ± 3.87	N/A	14.57 ± 18.23	26.49 ± 23.61	0.86 ± 1.77	3.48 ± 5.59	54.23 ± 30.76	60.64 ± 14.69

Note: CC: control condition; CG: compression garments; MG: medial gastrocnemius; LG: lateral gastrocnemius; SO: soleus; TA: tibialis anterior; RF: rectus femoris; VM: vastus medialis; VL: vastus lateralis; BF: biceps femoris; GM: Gluteus Maximus. The bold values indicate the data with significant differences and are intended to highlight them.

^a^ Significant difference between CG, and CC, *p* < 0.05.

### 3.4 Activation curves

**FIGURE 4:**
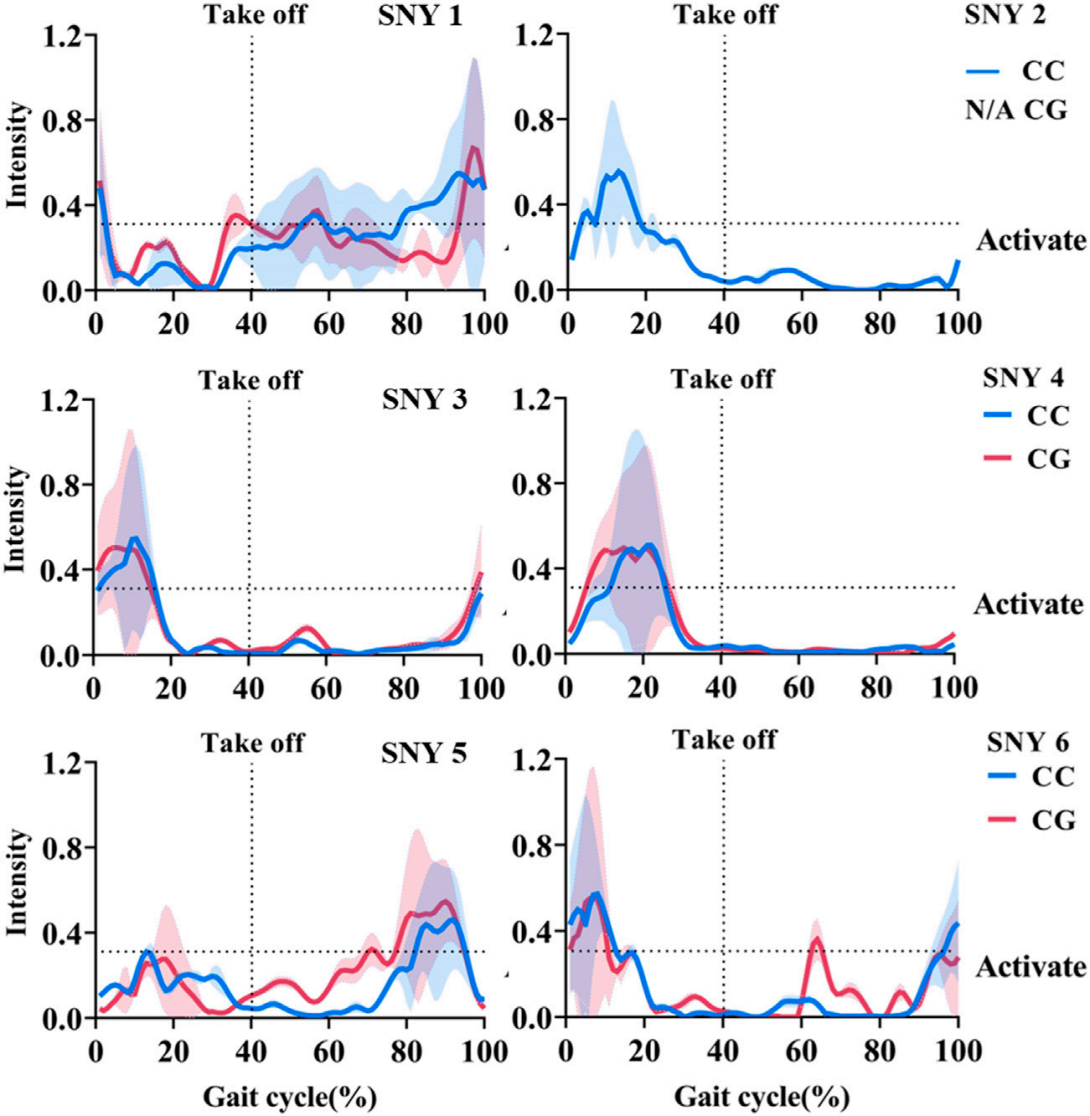
Average Activation Curve of SYN (1–6). Note: CC: Control Condition; CG: Compression Garments; SYN2 was not extracted in CG conditions; Shaded area is standard deviation of mean activation curve across participants.

Figure 4 shows the activation curves of these synergies during the gait cycle. The CGs did not affect the activation curve characteristics of SYN3, SYN5, and SYN6. However, the CGs condition shortened the activation duration of SYN1 by 40% (*p* < 0.001) and split its activation curve into two segments: one in the early swing phase and one before foot strike. CGs also lengthened the activation duration of SYN4 by 25% (*p* < 0.001). The peak activation time of SYN1 and SYN4 was not influenced by CGs. (Presented in Supplementary Table S3).

## 4 Discussion

The current study represents the first known investigation employing muscle synergy analysis to assess the efficacy of compression garments (CGs) on running biomechanics. The primary aim of this cross-sectional study was to investigate the acute effects of wearing CGs on running biomechanics and muscle synergies. Our findings supported our initial hypothesis that CGs altered the knee and hip joint kinematics, i.e., CGs significantly reduced hip and knee flexion at foot strike and increased the extension of hip and knee joint at take-off (Figure 1). However, the CGs did not affect the range of motion (ROM), moment and power of the three lower body joints during running. Notably, we extracted and classified six distinct synergies (SYN1-6) from the data of nine muscles we measured, and identified their respective functions and activation periods in the results section. Our findings on muscle synergies are consistent with the 4–6 synergies found in previous studies of running (Cappellini et al., 2006; Allen and Neptune, 2012; Mileti et al., 2020). Specifically, CGs modified the structure of the synergies, and induced alterations in the muscle activation weights and durations of some synergies. Our findings provide a more comprehensive understanding of the effects of CGs. Next, we delved into the kinematic alterations we have observed, adopting a muscle synergy framework to elucidate these changes and establish connections with the insights gleaned from prior studies.

Previous studies have investigated the effects of CGs on muscle activation. Hsu et al. (2020) observed that CGs led to a decrease in activation of the GM, RF, and semitendinosus (ST) during running. Similarly, Broatch et al. (2020) and Vercruyssen et al. (2017) reported a reduction in certain muscle activations attributed to CGs; however, no significant impact on running economy was observed. Despite the authors’ suggestion to consider higher-pressure CGs or alternative physiological indicators for future assessments, we contend that prior discussions have been constrained by the analytical method. The potential oversight lies in the fact that alterations in muscle activation may signify plausible changes in the neuromuscular control structure. During running, each muscular synergy may be dedicated to distinct functions, and these synergies are influenced by variables like running speed, ground conditions, and foot strike patterns (Nishida et al., 2017; Aoi et al., 2019; Yaserifar and Oliveira, 2023). This evidence suggests that muscle synergy is more sensitive to show adjustments in muscle function with some external influence and better permits explanation of neuromuscular performance adjustments. Consequently, our study examines the role of CGs through the lens of muscle function, specifically focusing on muscle synergy.

In our study, the CGs did not result in immediate alterations to kinetic parameters, including joint moment or power. This finding is consistent with our hypothesis and previous studies that during single or short-term exercise. For example, no differences were observed in 60 m and 400 m sprint time while wearing CGs (Doan et al., 2003; Engel, Holmberg, and Sperlich, 2016). Another study’s findings support this concept: Initially, CGs did not enhance sprint performance. However, with the ongoing sprint, CGs demonstrated an improvement in repeated sprint performance, particularly in the final 10 sprints (Born et al., 2014). The authors posit that this enhancement might be linked to the reduced hip flexion angle and increased step length during the sprints. Likewise, our investigation revealed that the immediate application of CGs led to changes in hip and knee joint kinematics (Figure 1), Based on the above evidence, we speculate that the effect of CGs indeed exists and influences performance through muscle management (i.e., the muscle synergy under the control of the nervous system). In review of previous research on CGs, these effects may demonstrate benefits in prolonged exercise (these benefits may be manifested in both kinetic (Born et al., 2014) and physiological (Engel, Holmberg, and Sperlich, 2016; Yang Y. et al., 2020) aspects). Therefore, the key point is that our findings of changes in muscle synergies may be able to bridge the gap between these evidences.

Kibushi, Moritani, and Kouzaki (2022) found that when the running stride length shortened, one synergy “disappeared”. They believed that the “disappeared” synergy was mainly responsible for the mechanical task of hip swing. When the stride length shortened, the hip swing was significantly reduced, so this synergy did not appear. Our study also found that after wearing CGs, the number of synergies decreased, SYN2 “disappeared”. The inability to observe SYN2 does not mean that it no longer exists, but rather that it cannot explain the original electromyographic signals, resulting in decomposition or fusion phenomena (Cheung et al., 2020), and the changes in SYN4 we found corroborate this statement: As shown in Figure 4, SYN2 activates earlier than SYN4. SYN4 activates earlier after SYN2 “disappears”, and the activation duration of it increased by 25% (Figure 4). Both SYN2 and SYN4 were mainly activated by calf muscles during the stance phase to perform propulsion function. Therefore, we believe that wearing CGs merged SYN2 into the functionally identical SYN4. Given that we did not observe any changes in lower limb kinetics after wearing CGs, we believe that this merging of specific muscle synergies is positive, completing the same movement task while reducing the number of managed muscle synergies. This merging of muscle synergies represents an increased level of running. A high-quality study found that running training led to merging of specific muscle synergies, with the extent of synergy merging increasing from sedentary subjects to elite runners (Cheung et al., 2020).

The effect of CGs on muscle synergies lacks references, but researchers generally believe that the pressure stimulation of CGs can provide “extra” proprioceptive information (Michael et al., 2014), and this extra “information” is considered to improve proprioceptive function and optimize motor performance. Early researchers found that CGs reduced joint angle repositioning error (Kraemer et al., 1998); Hooper et al. (2015) found that after familiarizing with CGs, the accuracy of hitting and slicing of high-level college golfers improved significantly. These studies suggest that external pressure may have some benefits for proprioception, posture control, and motor performance. Neurophysiological evidence supports the above observations: Lee, Kim, and Lee (2017) found that the pressure exerted by CGs on the skin and muscles changed the amplitude of related motor cortex potentials recorded by electroencephalogram. In our study, after wearing CGs, the weight of VL in SYN4 increased, and the activation duration increased by 25%, which may explain the larger peak knee extension angle at take-off (Figure 1). Increased hip extension during take-off is posited to contribute to enhanced propulsion in running (Charalambous et al., 2012), and is particularly advantageous for sprinting (van Ingen Schenau, de Koning, and de Groot, 1994). As previously elucidated, CGs not only facilitate the merging of SYN2 with SYN4, thereby streamlining the muscle activation process during propulsion phase which subsequently contribute to a more beneficial kinematic profile to the propulsive movement during running. This mechanism is conducive to improved running performance.

The SYN6 was mainly activated before foot strike to early stance phase, performing weight acceptance function, mainly dominated by RF, VM, VL, and GM activation. After wearing CGs, the activation weight of RF and VM decreased by 76% and 61%. Both of them are hip flexor muscles, we then speculate that the decrease in peak hip flexion angle and the increase of peak hip extension angle during the stance phase may be related to the decrease of flexor muscle activation weights in SYN6. On the other part, in a prior study, wearing CGs resulted in a decrease in thigh muscle activation during landing, while concurrently increasing the measured damping coefficient at the thigh (Deng et al., 2021). The author proposed that a portion of muscle activation during the landing phase serves the mitigating the soft tissue vibrations caused by the impact, and CGs may aid in this process, consequently leading to a reduction in thigh muscle activation. This conjecture supports in our observations of reduced thigh muscle activation weights in SYN6. In our study, SYN6 was indeed activated before foot-strike to afford the function of weight acceptance.

Based on the above evidence, we postulate that CGs may mainly affect the input of mechanoreceptors by external pressure, which in turn affects the motor control commands of CNS, and coordinates the joint movement by muscle synergies, making optimized movements possible. Although there is no direct neurophysiological evidence, our findings support these speculations. However, several limitations should be considered in the current study: first, only male participants were included in this study, and we suggest that female participants could be included in the future to further investigate the effects of CGs on muscle synergies, as well as differences between genders (Macchi et al., 2022; Santuz et al., 2022). Second, despite the patch electrodes were used to minimise extraneous pressure on the muscle belly, the application of electrode on the inside of CG still caused a slight elevation in pressure and a non-uniform pressure distribution, which is another limitation of this study. Last, the present study is a preliminary investigation, and only reports the immediate alterations associated with the utilization of CGs. The potential modifications in neuromuscular control strategies for repetitive power output or prolonged exercise remain to be explored.

## 5 Conclusion

The current study is the first to apply muscle synergy extraction to evaluate the effect of compression garments on running biomechanics. We found that wearing CGs altered the lower limb kinematics and associated muscle synergies during running. We extracted and classified six synergies (SYN1-6) during running and found that only five SYNs were observed after wearing CGs. The muscle activation weights, activation times and durations of these SYNs were altered by the CGs, and we speculated that these changes accounted for the reduced hip and knee flexion at foot strike, and the increased knee and hip extension at take-off. However, these changes did not directly enhance the moment or power output of the lower limb joints. The current study provides evidence for the influence of CGs on running mechanics and muscle activation patterns from a neuromuscular control perspective, and suggests further investigation of the effect of CGs on muscle synergy during prolonged exercise.

## Data Availability

The original contributions presented in the study are included in the article/Supplementary Material, further inquiries can be directed to the corresponding author.
