# Supplementary material for: Whole leg compression garments influence lower limb kinematics and associated muscle synergies during running
Source: Front Bioeng Biotechnol. 2024 Feb 20;12:1310464. doi: 10.3389/fbioe.2024.1310464 (PMC10912955; doi:10.3389/fbioe.2024.1310464)
Supplement: Supplementary file 1 [file Table1.docx]

Supplementary Materials

# Supplementary Figures and Tables

## Supplementary Tables

**Table 1. Effects of Compression Garment on Ankle, Knee and Hip Kinetics (Mean ± SD).**

| *Joint* |  | CC | CG | *p* | *Cohen’s d* |
| --- | --- | --- | --- | --- | --- |
| *Ankle* | *M_dor_* (N·m·kg^-1^) | 0.25±0.14 | 0.29±0.18 | 0.544 | 0.25 |
|  | *M_pla_* (N·m·kg^-1^) | -3.52±0.42 | -3.39±0.62 | 0.473 | 0.30 |
|  | *P_abs_* (W·kg^-1^) | -10.44±2.54 | -9.95±2.04 | 0.542 | 0.26 |
|  | *P_rel_* (W·kg^-1^) | 17.49±3.38 | 17.82±4.48 | 0.767 | 0.12 |
| *Knee* | *M_fle_* (N·m·kg^-1^) | -1.12±0.40 | -1.02±0.41 | 0.438 | 0.33 |
|  | *M_ext_* (N·m·kg^-1^) | 1.77±0.38 | 1.93±0.74 | 0.464 | 0.31 |
|  | *P_abs_* (W·kg^-1^) | -8.73±2.91 | -8.47±1.92 | 0.683 | 0.17 |
|  | *P_rel_* (W·kg^-1^) | 8.05±3.81 | 7.26±3.75 | 0.526 | 0.27 |
| *Hip* | *M_fle_* (N·m·kg^-1^) | 0.86±0.22 | 0.94±0.35 | 0.504 | 0.28 |
|  | *M_ext_* (N·m·kg^-1^) | -4.39±0.52 | -3.99±1.24 | 0.219 | 0.53 |
|  | *P_rel_* (W·kg^-1^) | 8.34±2.91 | 7.09±2.96 | 0.112 | 0.71 |
|  | *P_rel_* (W·kg^-1^) | -4.17±3.08 | -2.92±2.42 | 0.231 | 0.52 |
| *Leg* | *k* (BW/m) | 26.54±1.32 | 27.07±2.01 | 0.465 | 0.31 |
|  | *W* (J/kg) | 0.20±0.07 | 0.21±0.08 | 0.738 | 0.13 |

Note: CC: Control Condition; CG: Compression Garments; *M_dor_*: Peak dorsiflexion moment; *M_pla_*: Peak plantarflexion moment; *M_fle_*: Peak flexion moment; *M_ext_*: Peak extension moment; *P_abs_*: Peak power absorption; *P_rel_*: Peak power release; *k*: vertical stiffness of lower limb at weight acceptance; *W*: normalised vertical energy loss; BW: body weight normalised.

**Table 2 Weight of Muscles Activation in SYN (Mean ± SD).**

| Muscles (%) | SYN1 | | SYN3 | | SYN4 | | SYN5 | | SYN6 | |
| --- | --- | --- | --- | --- | --- | --- | --- | --- | --- | --- |
|  | CC | CG | CC | CG | CC | CG | CC | CG | CC | CG |
| MG | 0.86±2.00 | 2.04±3.61 | 8.83±9.75 | 6.86±7.17 | 66.25±24.96 | 74.85±25.26 | 3.73±4.74 | 4.35±7.36 | 3.95±4.83 | 6.90±10.80 |
| LG | 0.75±1.13 | 8.63±25.28 | **6.64±8.59** | **20.95±16.03*** | 58.11±24.62 | 55.82±18.90 | 4.19±7.15 | 5.20±6.90 | 12.87±13.99 | 16.40±6.71 |
| SO | 1.99±3.36 | 15.10±31.35 | 21.83±18.93 | 21.12±18.81 | 49.98±19.87 | 64.49±18.14 | 2.09±2.26 | 3.64±5.10 | 3.79±3.37 | 5.34±5.28 |
| TA | 87.42±20.43 | 89.60±15.74 | 5.01±8.31 | 3.42±4.10 | 10.82±17.60 | 9.75±9.48 | 13.41±6.37 | 13.85±11.80 | 7.12±5.67 | 10.21±8.81 |
| RF | 15.58±16.95 | 12.54±16.13 | 32.67±17.65 | 40.40±25.60 | 5.90±9.88 | 5.81±9.47 | 4.12±6.75 | 2.28±2.96 | **28.17±32.19** | **6.74±5.37*** |
| VM | 4.15±4.30 | 15.03±26.54 | 56.67±24.84 | 67.54±18.65 | 8.90±4.37 | 6.14±6.50 | 5.88±6.09 | 9.04±7.49 | **25.82±19.40** | **10.07±18.58*** |
| VL | 3.10±4.16 | 11.99±31.06 | 49.82±25.74 | 54.93±21.66 | **2.55±2.42** | **6.66±3.93*** | 3.45±3.15 | 7.21±9.05 | 28.67±23.41 | 17.09±21.59 |
| BF | 6.04±4.72 | 12.99±30.72 | 5.35±4.96 | 1.44±1.74 | 10.35±15.14 | 9.80±11.66 | 86.60±17.31 | 83.03±13.50 | 3.61±5.20 | 0.68±0.74 |
| GM | 9.08±7.46 | 15.06±20.78 | 14.57±18.23 | 26.49±23.61 | 0.86±1.77 | 3.48±5.59 | 7.23±8.36 | 11.64±11.45 | 54.23±30.76 | 60.64±14.69 |

Note: CC: Control Condition; CG: Compression Garments; MG: Medial Gastrocnemius; LG: Lateral Gastrocnemius; SO: Soleus; TA: Tibialis Anterior; RF: Rectus Femoris; VM: Vastus Medialis; VL: Vastus Lateralis; BF: Biceps Femoris; GM: Gluteus Maximus; ^*^: Significant difference between CG and CC, *p* < 0.05.

**Table 3 Parameters of SYN Activation Curve During Gait Cycle (100%)**

|  | SYN1 | | SYN2 | | SYN3 | | SYN4 | | SYN5 | | SYN6 | |
| --- | --- | --- | --- | --- | --- | --- | --- | --- | --- | --- | --- | --- |
|  | CC | CG | CC | CG | CC | CG | CC | CG | CC | CG | CC | CG |
| Duration (%) | **61.22±4.23** | **36.81±6.21*** | 15.15±3.78 | N/A | 17.61±3.99 | 18.91±4.17 | **20.93±4.10** | **24.30±3.65*** | 17.30±3.56 | 19.83±4.13 | 19.89±3.64 | 16.12±3.79 |
| Peak (*t_max_*) | 98.76±1.19 | 97.57±2.57 | 12.83±2.65 | N/A | 11.19±1.69 | 9.44±2.54 | 18.84±3.10 | 21.46±2.61 | 87.02±1.63 | 83.85±1.20 | 5.26±1.34 | 7.62±1.65 |
| Start *(t_start_*) | 42.31±1.60 | **39.01±1.05;**  **92.71±2.56** | 5.08±1.06 | N/A | 99.65±2.86 | 98.98±1.30 | 7.61±2.28 | 5.68±1.99 | 80.75±2.28 | 77.14±2.42 | 94.14±1.61 | 95.65±2.71 |
| End (*t_end_*) | 3.67±2.89 | **71.74±2.15;**  **3.03±1.99** | 19.98±2.49 | N/A | 16.62±2.77 | 16.35±2.23 | 26.75±1.44 | 28.48±2.33 | 96.22±1.93 | 95.71±2.39 | 13.94±1.02 | 11.84±1.41 |

Note: SYN1 showed a biphasic activation pattern under the CG condition, therefore *t_start_* and *t_stop_* of this phrase were not compared to those under other conditions. CC: Control Condition; CG: Compression Garments; ^*^: Significant difference between CG and CC, *p* < 0.05.
